# Single Versus Bilateral Internal Thoracic Artery Grafting in Patients on Chronic Dialysis

**DOI:** 10.3390/jcm14134451

**Published:** 2025-06-23

**Authors:** Ariel Farkash, Amit Gordon, Nadav Teich, Orr Sela, Mohammad Kakoush, Tomer Ziv Baran, Dmitry Pevni, Yanai Ben-Gal

**Affiliations:** 1 Department of Cardiothoracic Surgery, Tel Aviv Sourasky Medical Center, Faculty of Medicine, Tel Aviv University, Tel Aviv 6997801, Israel; arielfa@tlvmc.gov.il (A.F.); amitgor@tlvmc.gov.il (A.G.); nadavt@tlvmc.gov.il (N.T.); orrse@tlvmc.gov.il (O.S.); mohammadk@tlvmc.gov.il (M.K.); dimitrip@tlvmc.gov.il (D.P.); 2 Department of Epidemiology and Preventive Medicine, School of Public Health, Faculty of Medicine, Tel Aviv University, Tel Aviv 6997801, Israel

**Keywords:** CABG, BITA, dialysis, ESRD, renal, sequential, cardiac surgery

## Abstract

**Objective**: To evaluate the outcome of single vs. bilateral internal thoracic artery (SITA vs. BITA) revascularization in patients with multivessel coronary disease referred for coronary artery bypass graft (CABG) while on chronic dialysis. **Methods**: This retrospective analysis included all the patients with multivessel disease on chronic dialysis who underwent isolated CABG in our center during 1996–2021, utilizing SITA or BITA revascularization. We further matched the groups according to patient age and EuroSCORE II ±0.5. **Results**: Of the 7547 patients with multivessel disease who underwent CABG, 77 were on chronic dialysis. Of these, 2 had incomplete follow-up data, 58 underwent SITA, and 17 BITA revascularization. Comparing the SITA group with the BITA, the mean age was higher (67.8 vs. 58.6 years, standardized mean difference 1.035); the median (interquartile range) EuroSCORE II was higher (3.73 (1.78–6.23) vs. 1.78 (1.38–3.50), standardized mean difference 0.934); and comorbidities were more prevalent. Early mortality did not differ between the BITA and SITA groups in the unmatched cohort (11.8% vs. 15.5%, *p* > 0.999) or in the matched cohort (12.5% vs. 6.3%, *p* = 0.999). Other early adverse events such as early stroke, myocardial infarction, and bleeding requiring re-exploration were also similar. The median survival was 1.22 ± 0.5 years for the SITA and 5.64 ± 1.50 years for the BITA group. The respective five-year survival rates were 22.5 ± 5.9% and 58.35 ± 13.80%, *p* = 0.005. For the matched cohort, comprising 16 patient pairs, the five-year survival did not differ between the groups (27.8 ± 11.7% vs. 54.7 ± 14.7%, *p* = 0.258). In multivariable analysis, adjusted to EuroSCORE II and age, the hazard ratio (95% confidence interval) for BITA revascularization was insignificant, 0.638 (95% CI 0.25–1.62), *p* = 0.343. The hazard ratios for age and EuroSCORE II were 1.061 (95% CI 1.023–1.101), *p* = 0.002 and 1.155 (95% CI 1.070–1.246), *p* < 0.001. **Conclusions**: Despite a trend in favor of BITA utilization, no clear long-term survival benefit was demonstrated for BITA revascularization in patients on chronic dialysis after CABG.

## 1. Introduction

The number of individuals with kidney failure treated by dialysis in the US has risen, and the mortality rate for patients on hemodialysis has been cited as over 20% per year [1,2]. About half of this mortality is due to a cardiovascular cause; the yearly cardiovascular mortality rate is about 9% [3]. Renal dysfunction has been shown to be associated with accelerated atherosclerotic cardiovascular disease [4,5]. The aging of the general population, together with general medical advancements, has resulted in an increased incidence of cardiac surgery among individuals on dialysis [6,7]. End-stage renal disease (ESRD) treated by dialysis is generally a poor prognostic factor for coronary artery bypass grafting (CABG) [8,9,10]. Consequently, ESRD is included in several risk assessment tools for patients evaluated for surgical revascularization [8,11,12]. Nonetheless, a survival benefit of surgical revascularization over percutaneous intervention in the setting of renal dysfunction has been demonstrated in few studies [13,14].

Multi-arterial CABG, specifically using both mammary arteries, is the common revascularization strategy in our center. This practice, known as bilateral internal thoracic artery (BITA) grafting has demonstrated better long-term outcomes than single internal thoracic artery (SITA), in the general population [15,16,17] and among the subset of patients with renal impairment that do not require dialysis [18]. In a previous publication from our group, BITA grafting in non-dialysis patients with chronic renal failure was also associated with improved long-term outcomes [19].

Previous studies did not find a survival benefit following the use of both mammary arteries in dialysis patients [20,21]. In this report, we sought to examine the short- and long-term outcomes of BITA vs. SITA in patients with ESRD treated with dialysis.

## 2. Patients and Methods

### 2.1. Study Design and Population

The study population included all the patients with multivessel coronary disease who underwent isolated CABG in Tel Aviv Sourasky Medical Center during 1996–2021. Surgical procedure consisted of either BITA revascularization to the left coronary system or SITA revascularization to the left anterior descending artery, with or without additional grafts—primarily saphenous vein grafts to non-left anterior descending artery targets. For the current analysis, only patients with renal impairment requiring hemodialysis or peritoneal dialysis prior to surgical revascularization were included. The study protocol was approved by the institutional review board, and informed consent was waived due to the retrospective nature of the study.

At our institution, the choice between BITA and SITA grafting is primarily determined by the discretion of the operating surgeon. BITA revascularization is often employed even in high-risk patients [22,23], including those receiving hemodialysis. However, BITA grafting is generally avoided in patients with an elevated risk of sternal wound complications such as very elderly individuals, patients with chronic obstructive pulmonary disease, and women with obesity or diabetes mellitus [24,25]. Revascularization of the right coronary artery is typically performed using saphenous vein grafts, and all ITA grafts are harvested using the skeletonized technique. Further technical details of the BITA procedures performed at our center have been described in prior publications [24,26].

### 2.2. Data Collection and Definitions

Patient data were evaluated in accordance with EuroSCORE II clinical data definitions [12]. Early mortality was defined as death occurring within 30 days following surgery or during the index hospitalization. Cerebrovascular accident was defined as the occurrence of a new permanent neurological deficit confirmed by a computed tomographic demonstrating cerebral infarction. A deep sternal wound infection was defined as an infection involving the sternum or underlying tissues that necessitated re-sternotomy. Emergency procedures were defined as surgeries performed within 24 h of cardiac catheterization [27] or procedures indicated by the presence of acute evolving myocardial infarction (MI), pulmonary edema, or cardiogenic shock [28]. Early adverse outcomes included mortality, postoperative stroke, perioperative or early myocardial infection, a deep sternal wound infection, and re-operation for bleeding. Information on early outcomes was collected from discharge summaries, patient medical records, and departmental databases, while long term survival data were obtained from the Israeli National Registry.

### 2.3. Statistical Analysis

Categorical variables were presented as counts and percentages. Continuous variables were assessed for normality using histograms and reported as means with standard deviations or as medians with interquartile ranges based on distribution characteristics. Follow-up duration was calculated using the reverse censoring method. Comparisons of early outcomes between surgical groups were performed using Fisher’s exact test. Survival analysis was conducted using Kaplan–Meier curves in order to estimate survival rates and median survival times, with a log-rank test applied for group comparisons.

To evaluate associations between surgical strategy and mortality while controlling for potential confounders, multivariable Cox proportional hazards models were constructed. Three models were tested: first adjusting for age and sex, then adjusting for EuroSCORE II as a confounder, and finally adjusting for both age and EuroSCORE II.

Patients were matched based on EuroSCORE II, allowing a maximum absolute difference of 0.5. Standardized differences were calculated before and after matching to assess the balance between groups; values < 0.1 were considered negligible, while values between 0.1 and 0.2 indicated a small imbalance. A stratified Cox regression by matched pairs was used to compare survival outcomes in the matched cohort. To account for potential biases related to surgical experience, institutional learning curves, technological advancements, and changes in pharmacologic management, while ensuring comparable group sizes, an analysis was conducted by comparing periods before and after the year 2015.

All statistical tests were two-sided, with *p*-values < 0.05 considered statistically significant. Statistical analyses were performed using IBM SPSS for Windows, version 29 (IBM Corp., Armonk, NY, USA, 2023).

## 3. Results

### 3.1. Baseline Characteristics of the Unmatched and Matched Cohorts

During the years 1996–2021, 7547 patients were operated upon for isolated CABG, of whom 77 were being treated by chronic dialysis when they underwent the procedure. After excluding two patients with incomplete follow-up data, 75 patients were included in the final cohort. Most of the patients (*n* = 58, 77.3%) underwent SITA revascularization (SITA group), while in 17 patients (22.7%), both mammary arteries were used for surgical revascularization (BITA group). 

The baseline characteristics of the unmatched and matched cohorts are presented in Table 1. In the unmatched cohort, for the SITA compared to the BITA group, the mean age was older (67.8 vs. 58.6 years, standardized mean difference (SMD) 1.035), the EuroSCORE II was higher (3.73 vs. 1.78, SMD = 0.934), and a higher proportion had comorbidities. The latter included chronic obstructive pulmonary disease, diabetes mellitus, peripheral vascular disease, and low ejection fraction. Expectedly, saphenous vein grafting was used more often in the SITA group (81.0% vs. 41.2%, SMD = 0.896).

Due to the small cohort number, we performed a further sub analysis in which the matching was based on the patient’s EuroSCORE II ± 0.5 score difference and age. The matched cohort included 16 patients in each group. After matching, some baseline parameters still differed between the groups (Table 1). However, we believe that the most dominant parameters, which reflect the overall patients’ clinical status, are the EuroSCORE II level and patient age.

### 3.2. Early Outcomes

In the unmatched cohort, there was no significant difference in early mortality between the BITA and SITA groups (11.8% and 15.5%, respectively, *p* > 0.999). Also in the matched cohort, mortality was similar: one (6.3%) vs. two (12.5%, *p* = 0.999). For both the matched and unmatched cohorts, there were no significant differences between the BITA and SITA groups in all the early outcomes examined, including early strokes, MIs, and bleeding requiring re-exploration (Table 2).

### 3.3. Late Outcomes

**Figure 1:**
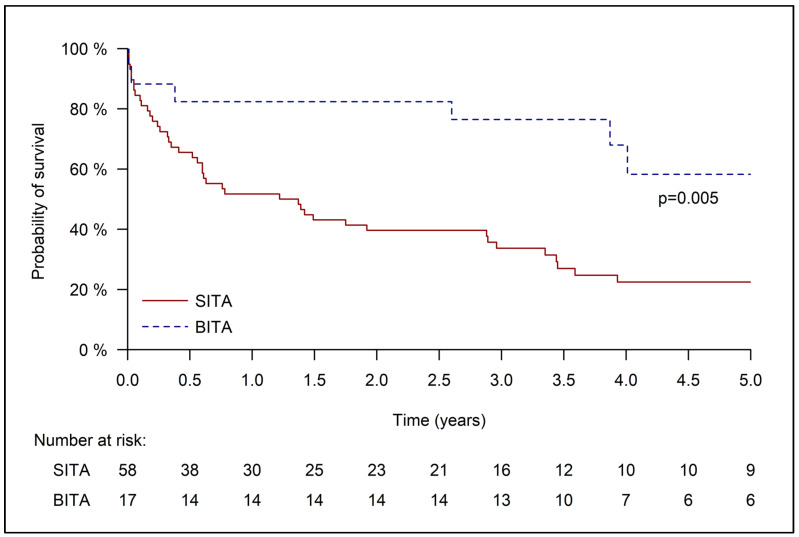
Kaplan–Meier curve for 5-year survival of the unmatched cohort.

As very few patients survive until the 10- and 20-years follow-up, we performed a five-year survival analysis. The median survival of the whole cohort was 1.92 +/− 0.73 years (1.22 +/− 0.5 for the SITA group and 5.64 +/− 1.5 years for the BITA group) (Figure 1). For the unmatched cohort, the five-year survival was worse for the SITA than the BITA group (22.5 ± 5.9% vs. 58.35 ± 13.8%, *p* = 0.005).

**Figure 2:**
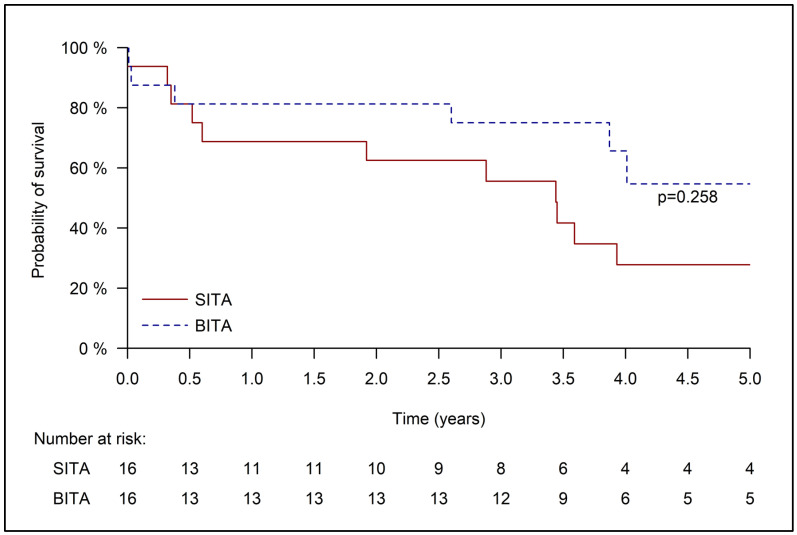
Kaplan–Meier curve for 5-year survival of the matched cohort.

In the matched cohort (Figure 2), the difference between the groups in five-year survival was not statistically significant (SITA 27.8 ± 11.7% and BITA 54.7 ± 14.7%, respectively, *p* = 0.258). The median survival for the SITA group was 3.44 +/− 0.5 years. The median survival for the matched BITA group was not reached, as more than half the patients survived more than five years.

In a univariate analysis, BITA revascularization showed a significant protective effect (hazard ratio [HR] 95% confidence interval (CI) 0.311 (95% CI 0.132–0.735), *p* = 0.008). In a multivariable analysis, adjusted to EuroSCORE II and age, the HR for BITA revascularization was insignificant, 0.638 (95% CI 0.25–1.62, *p* = 0.343). In contrast, both age (HR 1.061 (95% CI 1.023–1.101, *p* = 0.002) and EuroSCORE II HR 1.155 (95% CI 1.07–1.246, *p* < 0.001) were found to be significant. The other multivariable analyses (adjusted to EuroSCORE II alone and adjusted to age + sex) showed similar results.

## 4. Discussion

As the general population ages, an increasing proportion of patients with coronary artery disease who present for surgical revascularization suffer from reduced renal function [6,7,8]. Several studies described CABG as a superior treatment strategy in this patient population when compared to percutaneous intervention or medical therapy [13,14]. Still, reduced renal function and not only ESRD is a well-known risk factor in cardiac surgery and is included in several surgical revascularization risk assessment tools [8,9,10,11,12].

Among the various mechanisms that have been postulated for the negative impact of renal impairment is an increase in the severity and acceleration rate of atherosclerotic disease, the impairment of tissue quality, and unveiled uremia-related risk factors. Moreover, renal impairment is often an expression of other co-morbidities such as diabetes with end organ damage [5,29,30].

As the gold standard for surgical revascularization [15,16,17], BITA grafting has been associated with improved long-term survival but is considered a more demanding surgical strategy.

We previously reported a possible positive impact of BITA revascularization on the outcomes of patients with impaired renal function, during an earlier era (1996–2011) with no data regarding preoperative dialysis [19]. In the current report, we focused only on patients with ESRD who were on chronic dialysis treatment when referred to the CABG procedure. This study failed to demonstrate any early outcome advantage or a clear long-term survival benefit for BITA over SITA revascularization in patients with ESRD. In the unmatched cohort, five-year survival was better following the BITA procedure than SITA (58.3 ± 13.8% vs. 22.5 ± 5.9%, *p* = 0.005). However, as the characteristics of the groups differed considerably, this advantage in risk factors, together with other indistinctive clinical parameters, raises the probability of a selection bias and precludes reaching a reliable conclusion. In the matched cohort, we did not find any early or late (five-year) survival difference following BITA or SITA revascularization. For the respective groups, early mortality rates were 1 (6.3%) and 2 (12.5%), *p* = 0.999, and late survival rates were 54.7 ± 14.7% and 27.8 ± 11.7%, *p* = 0.258. 

The findings of our study are in concordance with previously published results on this topic. In a retrospective study, Nakayama et al. reported similar perioperative results for 25 patients on dialysis following BITA CABG and 52 patients with ESRD following SITA CABG [31]. Notably, they did not perform a mid- or long-term follow up. 

In a retrospective analysis of patients treated by hemodialysis, with a mean 3.3 years follow-up, Hachiro et al. [21] reported similar early mortality and midterm results between 86 patients who underwent BITA and 59 patients who underwent SITA revascularization. Of note, all the patients were operated upon using an off-pump technique, while this was used for only 24.1% of our patients who underwent BITA and 11.8% of those who underwent SITA revascularization. 

Among hemodialysis patients, Munakata et al. [32] compared 33 who underwent SITA grafting to 30 who underwent BITA grafting; 45% of the former and 33% of the latter were operated upon without cardiopulmonary bypass. In a long-term follow up (mean 4.2–4.4 years), no significant difference was noted between groups in overall or cardiac-related mortality. 

Among patients with ESRD, Kai et al. [33] found no significant difference in perioperative outcomes between the 76 patients who followed BITA and the 25 after SITA, during a median follow-up of 3.1 years. However, for the BITA group, they reported a trend toward better survival and freedom from cardiac mortality. 

A multicenter study by Gatti et al. [34] evaluated 105 patients on dialysis who underwent BITA grafting. Notably, the hospital mortality was twice the expected risk by EuroSCORE II (18.1% vs. 9.1% expected risk) and higher than was previously reported for BITA grafting in ESRD. With a mean follow-up of 3.8 years, the freedom from all cause death at five years was 68.1%. In propensity score matching, no difference was found between the 19 patients who underwent SITA and the 40 who underwent BITA revascularization, in risk-adjusted all-cause or cardiac death. Finally, Tam et al. [20] conducted a meta-analysis of patients with ESRD, which included five studies, with a total of 228 patients after BITA, and 200 after SITA. Differences were not found between the groups, in either short- or long-term mortality, at a mean follow-up of 3.7 years. 

Cardiac surgery in ESRD is often more complex due to typical clinical features and tissue changes, such as anemia, uremic pericarditis causing pericardial adhesions, fluid overload, vascular calcifications causing calcified aorta, and poor conduit quality [6,29,30]. Conduit selection for CABG in patients on dialysis is challenging, as utilization of both mammary arteries in these patients bears a questionable advantage considering their relatively short-term survival. Compared to arterial grafts, the general detriment of saphenous vein grafts is attributed to their late failure, five to ten years postoperatively. This well-known atherosclerosis process is probably accelerated in patients with renal dysfunction with reports of limited vein graft patency in ESRD patients compared to the general CABG population [6]. Of note is that dialysis patients rarely receive more than a single internal thoracic artery (ITA) graft, so data on the survival advantage of BITA grafting in this patient cohort remain limited [19]. In addition, using an ITA ipsilateral to the arteriovenous dialysis fistula might cause flow changes and a steal effect [35], especially while it is anastomosed to a left anterior descending territory with severe proximal stenosis and an extensive vascular bed [36]. As for radial artery grafts, their use is usually prohibited in CRF patients, due to the potential need for arteriovenous fistulas in future dialysis.

### Limitations

This study has limited statistical power due to the small sample size within the matched cohort. Matching was performed based on EuroSCORE II, a well-validated tool for assessing surgical risk. However, propensity score matching or inverse probability weighting could not be applied because of the limited number of BITA patients. In addition, the single-center retrospective design bares some inherent limitations. The selection of SITA vs. BITA strategy was based upon surgeons’ preferences, with no stringent guidelines to dictate either strategy. Consequently, a treatment allocation bias cannot be ruled out even after the attempt to perform age- and EuroSCORE II-based assessment. In addition, we did not differentiate between dialysis modalities as most studies reported a similar long-term survival between peritoneal dialysis and hemodialysis [37]. Data regarding the complete follow-up of post-discharge renal function and of the major adverse cardiac events after index hospitalization were not available. Some angiographic characteristics (lesion-type, severity, and syntax score) that might have reinforced or refuted our clinical observations were also unavailable. As mentioned, the location of the AV fistula side might affect graft patency, but unfortunately, data regarding fistula side and graft patency were unavailable. Finally, we had no data regarding the duration of dialysis prior to surgery and no information regarding patients who underwent kidney transplantation after their cardiac surgery, which could have an effect on a patient’s survival.

## 5. Conclusions

In conclusion, this study did not demonstrate an early or late benefit for using BITA grafting in patients with ESRD. The patients chosen for BITA grafting were younger and healthier and had lower surgical risk. Still, this “real life” setup demonstrates a possible long-term survival advantage in using BITA for selected patients with ESRD. We believe that the potential benefit of multi-arterial revascularization manifested in the general population should be weighed against the expected poor prognosis in ESRD for each individual patient. Additional studies are required to specify the characteristics of the ESRD population that might benefit from surgical revascularization that deploys both internal thoracic arteries.

## Figures and Tables

**Table 1:** Preoperative and intraoperative characteristics of patients on chronic dialysis. The data are presented for the unmatched and matched cohorts.

	All	Unmatched Cohort *n* (%)			Matched Cohort *n* (%)		
		SITA	BITA	SMD	SITA	BITA	SMD
	*n* = 75	*n* = 58	*n* = 17		*n* = 16	*n* = 16	
Male	59 (78.7%)	45 (77.6%)	14 (82.4%)	0.119	16 (100%)	14 (87.5%)	0.535
Age (years), mean (SD)	65.69 (10.07)	67.78 (9.69)	58.56 (8.05)	1.035	63.88 (10.95)	59.43 (7.43)	0.476
Age ≥ 70	29 (38.7%)	28 (48.3%)	1 (5.9%)	1.085	6 (37.5%)	1 (6.3%)	0.816
NIDDM	32 (42.7%)	26 (44.8%)	6 (35.3%)	0.195	6 (37.5%)	6 (37.5%)	0.000
IDDM	17 (22.7%)	14 (24.1%)	3 (17.6%)	0.160	6 (37.5%)	2 (12.5%)	0.603
DM	46 (61.3%)	38 (65.5%)	8 (47.1%)	0.379	12 (75%)	7 (43.8%)	0.671
COPD	11 (14.7%)	10 (17.2%)	1 (5.9%)	0.361	4 (25.0%)	1 (6.3%)	0.535
CHF	31 (41.3%)	23 (39.7%)	8 (47.1%)	0.150	6 (37.5%)	7 (43.8%)	0.128
Recent MI	17 (22.7%)	17 (29.3%)	0 (0%)	0.911	5 (31.3%)	0 (0%)	0.953
Old MI	23 (30.7%)	19 (32.8%)	4 (23.5%)	0.206	6 (37.5%)	4 (25.0%)	0.272
Acute MI	20 (26.7%)	15 (25.9%)	5 (29.4%)	0.079	3 (18.8%)	5 (31.3%)	0.292
MI	43 (57.3%)	34 (58.6%)	9 (52.9%)	0.115	8 (50.0%)	9 (56.3%)	0.125
Unstable angina pectoris	62 (82.7%)	48 (82.8%)	14 (82.4%)	0.011	12 (75.0%)	13 (81.3%)	0.152
EF < 30%	15 (20.0%)	13 (22.4%)	2 (11.8%)	0.286	3 (18.8%)	1 (6.3%)	0.385
Intra-aortic balloon pump	5 (6.7%)	4 (6.9%)	1 (5.9%)	0.041	1 (6.3%)	1 (6.3%)	0.000
Emergent operation *	33 (44.0%)	25 (43.1%)	8 (47.1%)	0.080	2 (12.5%)	7 (43.8%)	0.741
Redo	1 (1.3%)	0 (0%)	1 (5.9%)	0.354	0 (0%)	1 (6.3%)	0.365
PVD	26 (34.7%)	24 (41.4%)	2 (11.8%)	0.712	7 (43.8%)	2 (12.5%)	0.741
Left main disease	25 (33.3%)	17 (29.3%)	8 (47.1%)	0.372	3 (18.8%)	8 (50.0%)	0.697
Prior PCI	33 (44%)	26 (44.83%)	7 (41.18%)	0.074	7 (43.8%)	7 (43.8%)	0.000
EuroSCORE II, median (IQR)	3.15 (1.78–6.23)	3.73 (1.95–7.1)	1.78 (1.38–3.5)	0.934	1.89 (1.51–3.08)	1.68 (1.37–3.13)	0.123
Bypass number ≥ 3	41 (54.67%)	30 (51.72%)	11 (64.71%)	0.266	9 (56.3%)	11 (68.8%)	0.260
Sequential anastomoses	33 (44%)	24 (41.4%)	9 (52.9%)	0.233	8 (50%)	9 (56.3%)	0.125
SVG	54 (72%)	47 (81%)	7 (41.2%)	0.896	13 (81.3%)	7 (43.8%)	0.840
GEA	6 (8%)	4 (6.9%)	2 (11.8%)	0.168	2 (12.5%)	2 (12.5%)	0.000
Right system revascularization	33 (44%)	26 (44.8%)	7 (41.2%)	0.074	9 (56.3%)	7 (43.8%)	0.252
OPCAB	16 (21.3%)	14 (24.1%)	2 (11.8%)	0.327	6 (37.5%)	2 (12.5%)	0.603
Operated after the year 2015	36 (48%)	28 (48.3%)	8 (47.1%)	0.024	6 (37.5%)	7 (43.8%)	0.128

SITA: single internal thoracic artery; BITA: bilateral internal thoracic artery; SD: standard deviation; NIDDM: non-insulin-dependent diabetes mellitus; IDDM: insulin-dependent diabetes mellitus; DM: diabetes mellitus;COPD: chronic obstructive pulmonary disease; CHF: congestive heart failure; MI: myocardial infarction; EF: ejection fraction; PVD: peripheral vascular disease; PTCA: percutaneous transluminal coronary angioplasty; IQR: interquartile range; SMD: standardized mean difference; SVG: saphenous vein graft; GEA: gastroepiploic artery graft; OPCAB: off-pump coronary artery bypass. * Defined as an operation performed within 24 h of catheterization or in patients with evident preoperative acute or evolving MI, pulmonary edema, or cardiogenic shock.

**Table 2:** Early outcomes of patients on chronic dialysis.

	All	Unmatched Cohort *n*(%)		
		SITA	BITA	*p* Value
	*n* = 75	*n* = 58	*n* = 17	
Early mortality	11 (14.7%)	9 (15.5%)	2 (11.8%)	>0.999
Deep infection	4 (5.3%)	4 (6.9%)	0 (0%)	0.568
Post CVA	5 (6.7%)	5 (8.6%)	0 (0%)	0.582
Perioperative MI	3 (4.0%)	2 (3.4%)	1 (5.9%)	0.543
Revision for bleeding	4 (5.3%)	3 (5.2%)	1 (5.9%)	>0.999

SITA—single internal thoracic artery; BITA—bilateral internal thoracic artery; CVA—cerebrovascular accident; MI—myocardial infarction.

## Data Availability

The datasets generated and analyzed during the current study are subject to restrictions. Due to ethical and legal considerations imposed by the Institutional Review Board of Tel Aviv Sourasky Medical Center, the data cannot be shared publicly in order to protect patient confidentiality. Requests for access to the data may be directed to Dr. Shmuel Kivity, Chairman of the Tel Aviv Sourasky Medical Center Institutional Review Board (IRB)/Ethics (Helsinki) Committee at allergy@tlvmc.gov.il.

## Abbreviations

The following abbreviations are used in this manuscript:

SITA	Single internal thoracic artery
BITA	Bilateral internal thoracic artery
CABG	Coronary artery bypass graft surgery
ESRD	End stage renal disease
